# Sex Differences in Countermovement Jump Phase Characteristics

**DOI:** 10.3390/sports5010008

**Published:** 2017-01-19

**Authors:** John J. McMahon, Sophie J. E. Rej, Paul Comfort

**Affiliations:** 1 Directorate of Sport, Exercise and Physiotherapy, University of Salford, Frederick Road, Salford, Greater Manchester, M6 6PU, UK; p.comfort@salford.ac.uk; 2 Department of Sport and Exercise Sciences, University of Chichester, Chichester, West Sussex, PO19 6PE, UK; srej1@stu.chi.ac.uk

**Keywords:** Gender Comparison, force-time, power-time, temporal phase analysis, neuromuscular function

## Abstract

The countermovement jump (CMJ) is commonly used to explore sex differences in neuromuscular function, but previous studies have only reported gross CMJ measures or have partly examined CMJ phase characteristics. The purpose of this study was to explore differences in CMJ phase characteristics between male and female athletes by comparing the force-, power-, velocity-, and displacement-time curves throughout the entire CMJ, in addition to gross measures. Fourteen men and fourteen women performed three CMJs on a force platform from which a range of kinetic and kinematic variables were calculated via forward dynamics. Jump height (JH), reactive strength index modified, relative peak concentric power, and eccentric and concentric displacement, velocity, and relative impulse were all greater for men (*g* = 0.58–1.79). Relative force-time curves were similar between sexes, but relative power-, velocity-, and displacement-time curves were greater for men at 90%–95% (immediately before and after peak power), 47%–54% (start of eccentric phase) and 85%–100% (latter half of concentric phase), and 65%–87% (bottom of countermovement and initial concentric phase) of normalized jump time, respectively. The CMJ distinguished between sexes, with men demonstrating greater JH through applying a larger concentric impulse and, thus, achieving greater velocity throughout most of the concentric phase, including take-off.

## 1. Introduction

The countermovement jump (CMJ), performed either with or without an external load, is commonly used both as part of athlete training programs to promote the development of lower body power [1,2,3] and athlete testing batteries to provide insight into neuromuscular function [4,5] and fatigue [6,7,8]. Additionally, the CMJ has frequently been used to identify age [4,9,10] and sex [11,12,13] differences in a range of performance variables to inform appropriate training prescription. In terms of the latter, many studies have showed that men jump higher than women [14,15,16], even when resistance-/jump-trained male and female athletes have been compared [11,12,13,17]. Several attempts to explain sex differences in the CMJ height of athletes have been made by analyzing CMJ force- and power-time records but with mixed results. For example, one study attributed sex differences in CMJ height to men’s ability to demonstrate larger relative (to body mass) peak concentric force, along with greater absolute and relative mean eccentric rate of force development (RFD) [12]. Contrastingly, however, an earlier study found no sex differences in CMJ absolute mean eccentric RFD or movement time [11]. Similarly, other studies reported no sex differences in relative peak concentric/propulsive force [13,17] and absolute mean eccentric RFD calculated for the CMJ [13]. The latter study did, however, report that relative peak concentric power and eccentric and concentric impulse generated in the CMJ was greater for men [13], whereas the former study found relative peak propulsive power and peak RFD to be similar between sexes, but propulsive impulse to be greater in men [17].

There are several possible reasons for the equivocal reports into the sex differences (or, indeed, lack thereof) in the CMJ force- and power-time records of athletes mentioned above [11,12,13,17], with the main reason being the inconsistent, and sometimes inaccurate, analyses of the different phases of the CMJ. For example, Riggs and Sheppard [17] reported peak variables (force, power, and RFD) and impulse in the propulsive phase of the CMJ but, within their methods, incorrectly defined this as the combined eccentric (sometimes termed “braking”) and concentric (sometimes termed “propulsive”) phases; therefore, it is not clear which specific peak values, eccentric or concentric, were reported [17]. The mean eccentric RFD and eccentric impulse reported by Rice et al. [13] actually included part of the unweighting phase of the CMJ. Furthermore, the impulse calculations adopted by both studies did not include the deduction of bodyweight [13,17], despite bodyweight likely being significantly different between sexes, thus advocating the calculation of net impulse for a fairer group comparison. The opposing results pertaining to correctly calculated mean eccentric RFD as a discriminator of CMJ performance between sexes [11,12] might be due the high variability associated with this measurement [18]. Neither study [11,12] reported reliability or variability statistics for mean eccentric RFD but the standard deviation was approximately half the mean value for the women [11] and the combined value for men and women [12] tested in these studies, suggesting that this variable demonstrated high between-subject variability. Therefore, mean eccentric RFD is probably not sensitive enough to detect ‘true’ sex-related CMJ performance differences.

The sex differences in relative peak concentric force reported by Laffaye et al. [12] could have been influenced by sex differences in negative center of mass (COM) displacement (i.e. squat depth) during the CMJ [18,19], but this was not considered by the authors, which makes conclusions difficult to ascertain. Although one would not expect squat depth in the CMJ to be standardized, as this would alter the subjects’ natural jump strategy, reporting changes in COM displacement during the CMJ would at least help to explain why sex differences or similarities in CMJ kinetics and other kinematic variables (e.g., velocity) are seen [18,19]. Unfortunately, only the study by Rice et al. [13] reported negative COM displacement for men and women finding no significant differences between sexes, despite men showing ~4 cm greater negative COM displacement. This may possibly be due to the small sample size for each group (*n* = 8) being insufficient enough to detect significant differences, but other work has shown that women adopt a shallower range of motion than men during the CMJ [20]. Rice et al. [13] did not, however, report positive COM displacement between the onset of the concentric phase and take-off which can also differ between groups and influence concentric velocity and, thus, concentric impulse and power [4]. Velocity was also not reported in any of the aforementioned studies [11,12,13,17] which omits part of information needed to explain why relative power (which is a product of force and velocity) was greater for men throughout a large portion of the concentric phase of the CMJ (including peak power) in the study conducted by Rice et al. [13], even when subjects were matched for relative strength levels.

As discussed above, there are several methodological limitations to previous studies that have attempted to explain kinetic and kinematic sex differences in the CMJ performances of male and female athletes [11,12,13,17]. Reporting gross CMJ variables alone is also considered to be a limitation due to this approach only providing insight into a small part of the CMJ performance, with recent work advocating the inclusion of a temporal phase analyses, alongside gross calculations, to provide a more detailed understanding CMJ performance [6]. This approach was included by Rice et al. [13] but, as mentioned earlier, additional temporal phase characteristics and gross variables, based on correct analysis procedures, could have been included in this work to provide a more comprehensive understanding of the reported sex differences in CMJ performance. The aim of this study was, therefore, to explore sex differences in CMJ phase characteristics by comparing force-, power-, velocity-, and displacement-time curves and several key gross variables attained by male and female athletes. It was hypothesized that men would jump higher than women due to attaining greater concentric velocity, as achieved by greater COM displacement within a similar movement time and, thus, greater relative concentric impulse and power.

## 2. Materials and Methods

### 2.1. Subjects

Fourteen female regional netballers (age 20 ± 2.3 years, height 1.68 ± 0.05 m, body mass 66 ± 7.4 kg) and fourteen male professional academy rugby league players (age 19 ± 1.3 years, height 1.82 ± 0.04 m, body mass 88 ± 8.8 kg) participated in this study. Subjects attended a single testing session scheduled at the same time of day in a laboratory setting. Written informed consent, or parental assent where appropriate, was provided prior to testing and the study was pre-approved by the institutional ethics committee.

### 2.2. Procedures

Following a brief dynamic warm-up, subjects performed three CMJs (interspersed with one minute of rest) to a self-selected depth [11,12,18]. Subjects were instructed to perform the CMJs as fast and as high as possible, whilst keeping their arms akimbo. Any CMJs that were inadvertently performed with the inclusion of arm swing or tucking of the legs during the flight phase of the jumps were omitted and additional CMJs were performed after one minute of rest [18].

### 2.3. Data Collection

All CMJs were recorded at 1000 Hz via a Kistler type 9286AA force platform and Bioware 5.11 software (Kistler Instruments Inc., Amherst, NY, USA). Participants stood still for the initial one second of the data collection period [15,21] to allow for the determination of body weight post-testing. The raw vertical force-time data for each CMJ trial were exported and analysed using a customized Microsoft Excel spreadsheet (version 2016, Microsoft Corp., Redmond, WA, USA). 

### 2.4. Data Analysis

**Figure 1:**
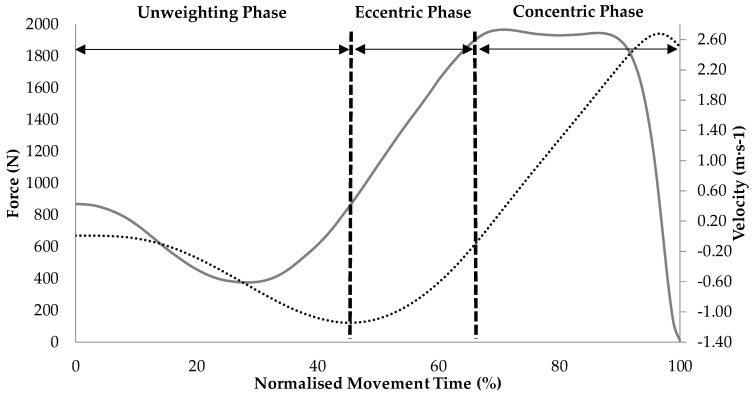
Countermovement jump phase interpretation based on force-time (grey solid line) and velocity-time (black dotted line) curve data (data represents the pooled mean of men’s force- and velocity-time curves).

Instantaneous COM velocity was calculated by dividing vertical force (excluding body weight) by body mass and then integrating the product using the trapezoid rule. Instantaneous power was calculated by multiplying vertical force and velocity data at each time point and COM displacement was determined by twice integrating vertical force data [21]. The start of CMJ was identified in line with current recommendations [18]. The eccentric phase of the CMJ was defined as occurring between peak negative and zero COM velocity. The concentric phase of the CMJ was defined as occurring between the instant at which COM velocity exceeded 0.01 m·s^−1^ and the instant of take-off (defined as the instant when vertical force was less than five times the standard deviation of the residual force [21]). The interpretation of the CMJ force-time curves attained in this study are in line with a recent study from our lab [18], and can be seen in Figure 1.

Eccentric and concentric peak force, peak power, and peak velocity were defined as the maximum values attained during the eccentric and concentric phases of the jump. Impulse was calculated during both the eccentric and concentric phases of the jump as the area under the net force-time curve (excluding body weight) using the trapezoid rule [19]. Leg stiffness was calculated as the ratio between peak eccentric force and eccentric COM displacement [22]. All kinetic data were divided by body mass to allow for a normalized comparison of these data between sexes. Jump height was derived from the vertical velocity at take-off [15]. A modified reactive strength index (RSImod) was calculated as the jump height divided by movement time [23].

The temporal phase analysis of the CMJ trials was conducted by modifying each subject’s force-, velocity-, power-, and displacement-time curves from the start of the CMJ through to take-off so that they each equaled 500 samples [18,24]. This was achieved by changing the time delta between the original samples (e.g., original number of samples/500) and subsequently re-sampling the data [18,24]. This resulted in an average sample frequency of 683 ± 112 and 620 ± 63 for women’s and men’s data, respectively, and allowed the averaged curve of each variable to be expressed over a percentage of time (e.g., 0%–100% of movement time which was defined the time from the onset of the unweighting phase to take-off). 

### 2.5. Statistical Analysis

For each gross measure the mean output of the three CMJ trials was taken forward for statistical analysis. All data satisfied parametric assumptions, as determined through the Shapiro-Wilk test, except movement time and peak eccentric power. Mean differences in each parametric variable derived for women and men were, therefore, compared using independent *t*-tests, whereas eccentric phase time and peak eccentric power were compared between sexes via the Mann-Whitney U test. A two-way random-effects model intraclass correlation coefficient (ICC) was used to determine the relative between-trial reliability of each gross variable. The ICC values were interpreted according to previous research where values ranging from 0.40 to 0.75 are considered good, and those over 0.75 are considered excellent [25]. Independent *t*-tests, the Mann-Whitney U test, and ICCs were performed using SPSS software (version 20; SPSS Inc., Chicago, IL, USA) with the alpha level set at *p* ≤ 0.05. Absolute between-trial variability of each gross variable was calculated using the coefficient of variation (calculated in this study as the typical error expressed as a percentage of the mean) expressed as a percentage (%CV). A CV of ≤10% was considered to be reflective of acceptable variability in line with previous recommendations [26]. Effect sizes were calculated using the Hedges’ *g* method to provide a measure of the magnitude of the differences in each gross variable noted between sexes and they were interpreted in line with previous recommendations which defined values of <0.35, 0.35–0.80, 0.80–1.5, and >1.5 as trivial, small, moderate, and large, respectively [27]. Likely sex differences in force-, velocity-, power-, and displacement-time curves were determined by plotting the time-normalized average curves for each cohort along with the corresponding upper and lower 95% confidence intervals (CI) to create upper and lower control limits and identifying non-overlapping areas [28]. 

## 3. Results

Except for movement time, which showed good between-trial reliability, all other variables demonstrated excellent between-trial reliability (Table 1). Furthermore, all variables showed acceptable between-trial variability apart from leg stiffness (Table 1). Men jumped significantly higher than women and demonstrated significantly greater RSImod values despite movement time being similar between sexes (Table 1). Within a similar movement time, men displayed significantly greater eccentric and concentric COM displacement, which resulted in a significantly greater peak velocity attained in each of these phases (Table 1). Relative peak eccentric and concentric force was similar between sexes, as were leg stiffness and relative peak eccentric power (Table 1). Relative peak concentric power was, however, significantly greater for men, as were relative eccentric and concentric impulse (Table 1). 

**Figure 2:**
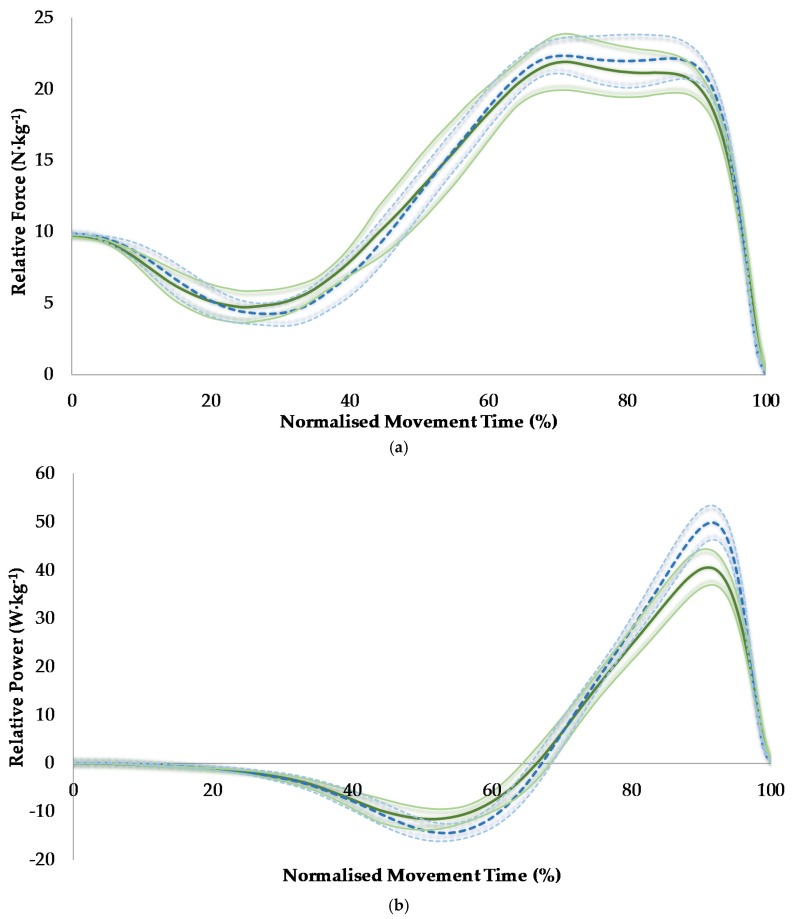

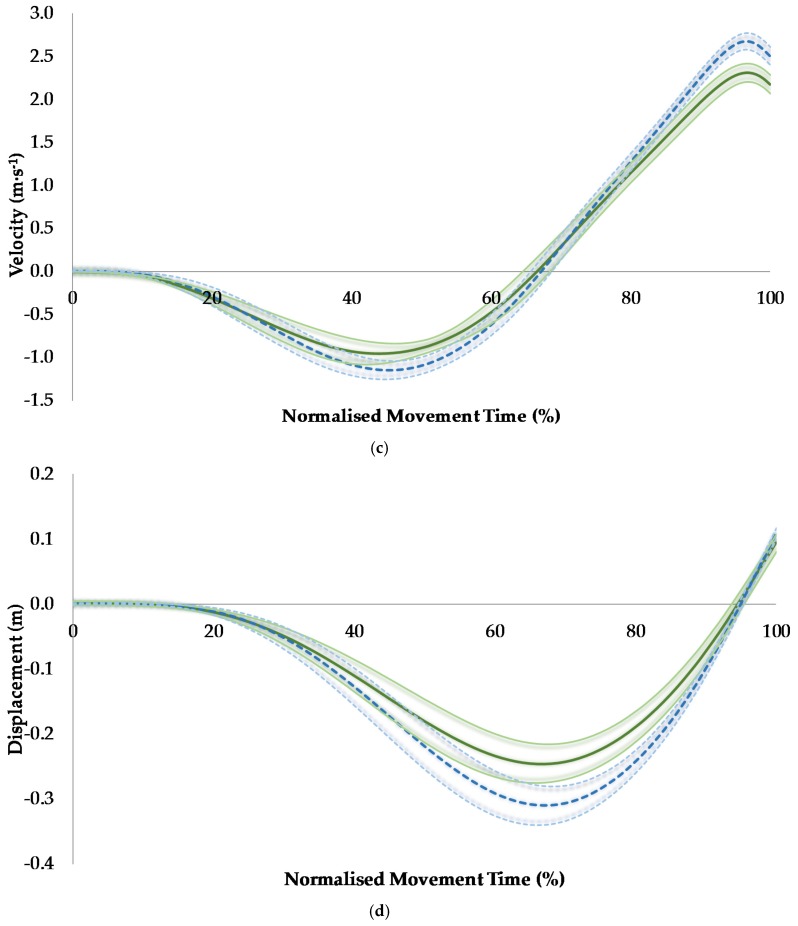
A comparison of countermovement jump force-time (**a**); power-time (**b**); velocity-time (**c**); and displacement-time (**d**) curves between women (green solid line) and men (blue dotted line) along with 95% confidence intervals.

The results of the temporal phase analysis showed that relative force was similar between sexes, but relative power was greater for men between 90% and 95% of the movement which corresponded to immediately before, during, and immediately after the attainment of peak concentric power (Figure 1). Velocity was greater for men, between 47%–54% and 85% and 100% of the movement, which corresponded to immediately after the onset of the eccentric phase and the latter half of the concentric phase of the jump, respectively (Figure 2). COM displacement was lower for men between 65% and 87% of the movement, which corresponded to the data points recorded just before the end of the eccentric phase and continued through approximately the first half of the concentric phase of the jump (Figure 2).

## 4. Discussion

The aim of this study was to explore sex differences in CMJ phase characteristics. Firstly, men jumped ~24% higher than women in line with the hypothesis and the range of ~25%–27% reported in similar studies [11,12,13]. It is known that jump height is determined by concentric net impulse [19], as a larger concentric net impulse will displace a given mass at a higher velocity based on the impulse-momentum relationship and velocity at take-off dictates jump height. As velocity is defined as displacement divided by time, the present results also show that men achieved a greater jump height by displacing their COM more than women did during the ground contact phase of the jump, but within a similar time (Table 1), thus attaining greater velocity throughout most of the concentric phase (including peak velocity) and at take-off (Figure 2). The sex differences in the relative power-time curve immediately before, during, and immediately after the attainment of peak concentric power (Figure 2) is in line with the results reported in a recent study [13]. The present study revealed that the increased relative power observed for men during this part of the CMJ is attributable to increased velocity during the latter half of the concentric phase of the jump (Figure 2), as the relative force-time curves were similar between sexes throughout the jump (Figure 2).

The finding that relative peak force attained in both the eccentric and concentric phases of the jump, as well as the force-time signature recorded throughout the jump, did not discriminate between men and women is in line with similar work [13,17]. The reason for similar forces demonstrated by both sexes is perhaps reflective of the different jump strategies employed by each group. For example, as mentioned earlier, women demonstrated less COM displacement during the eccentric and concentric phases of the jump which is reflective of a stiffer lower limb strategy. Indeed, leg stiffness was the only variable which showed a higher mean value for women, although there was no statistically significant difference in leg stiffness between sexes, probably owing to the large variability of this measurement (Table 1), although the associated effect size approached a moderate value (*g* = 0.71). Indeed, it has previously been described that women and men adopt differential leg stiffness strategies during jumping tasks [29]. Adopting a stiffer leg strategy in the CMJ acts to increase peak force [30] and so the similar force values reported in the present study may not have occurred if COM displacement was matched (at least relative to standing COM height) between sexes. Changing one’s preferred CMJ depth will change the natural jump strategy employed and the resultant output (i.e., jump height), which is why studies typically involve subjects squatting to their preferred depth during CMJ testing [11,12,13,17], but this alternate approach (i.e. matching squat depth) may warrant future investigation based on the present findings. The fact that the men and women tested in the present study were from different sports (i.e., rugby league and netball, respectively) may have influenced the natural jump strategy employed by each cohort [12], despite the trends reported here echoing those observed in studies which compared sexes from the same sport [13,17], thus, it may be prudent to match the sport as well as the squat depth in future similar work.

In contrast to the present and abovementioned studies [13,17], one earlier study reported relative peak concentric force to be larger for males, who jumped ~26% higher than their female counterparts [12]. Unfortunately, squat depth (i.e. eccentric COM displacement) was not reported in this study, so the leg stiffness strategy employed during the CMJ by the subjects tested is unclear. Mean eccentric RFD was greater for the men [12], suggesting that they adopted higher leg stiffness. A stiffer leg strategy is associated with shorter movement times [31,32] in addition to larger forces [30,31,32] which results in the production of an impulse that is characterized by a large force (i.e., tall) and short time (i.e., thin), which is beneficial for most sporting tasks (e.g., sprinting). Movement times were statistically similar in the present study and in two of aforementioned studies [11,12] but, in each of the latter studies, the mean movement time was greater for men whereas the opposite was noted in the present study (Table 1). This suggests that the men tested in the current study achieved an impulse that was longer in duration rather than larger in force, which is perhaps not a beneficial jump strategy for this cohort, in light of movements in sport mostly being time constrained. Even though the men tested in the current study achieved higher RSImod values than the women (Table 1), this was due to the men jumping higher rather than jumping with shorter movement time. Jumping higher and with a shorter movement time should be the aim for those whose sports would benefit from high reactive strength capacity, as this would produce the preferential tall- and thin-style of impulse mentioned earlier. This point also highlights that RSImod should be decompartmentalized to illustrate a jump height or movement time preponderance, as this would help to direct training priorities. 

Although men jumped higher than women in this study, both sexes jumped lower than the athletes tested in previous studies, which reported a CMJ height range of ~36–45 cm and ~45–60 cm for women and men, respectively [11,12,13,17]. It is probable, therefore, that both groups tested in the present study would benefit from becoming stronger, based on the notion that strength correlates highly with vertical jump height [33,34]. Increasing strength capacity should then result in increased ‘height’ of the concentric impulse generated due to increased force production. Increasing strength capacity through resistance training should also improve eccentric force- and power-time characteristics of the CMJ [1,2,3,35] which should subsequently improve CMJ height by improving stretch-shortening cycle function. Resistance training should also act to increase muscle size/mass and muscle-tendon stiffness [36,37,38] which are two factors that have been shown to be reduced in women [39,40] and have been related to the CMJ height deficiencies [41,42,43]. Although matching relative strength capacity did not negate all sex differences in CMJ kinetics observed in a recent study [13], this may have been due to differential jump strategies employed (e.g., squat depth), as mentioned earlier. Matching relative strength and squat depth in the CMJ between sexes might, therefore, yield more valuable information pertaining to this topic and, thus, should be considered as a future research avenue. Additionally, matching jump height between sexes in future studies might also help to reveal ‘true’ sex differences in CMJ kinetics and kinematics or, indeed, highlight sex-independency during this task.

## 5. Conclusions 

The CMJ distinguished between sexes, with men demonstrating greater jump height through applying a larger concentric impulse and, thus, achieving greater velocity throughout most of the concentric phase and at take-off. The larger concentric impulse and velocity achieved by men was attributed to them demonstrating larger COM displacement during the unweighting/eccentric phase of the jump (i.e. greater squat depth), which subsequently enabled them to achieved greater COM displacement during the concentric phase of the jump, but with a similar movement time to women. Women may, therefore, benefit from demonstrating a more compliant leg strategy during the CMJ, if increasing jump height is a desired outcome, but they should still aim to perform the countermovement in as short a time as possible to avoid reducing force production. This should enable women to achieve a greater concentric impulse that is force rather than time dominant, which is important for most athletes whose sport is characterized by high force/short contact time-focused tasks.

## Figures and Tables

**Table 1:** A sex comparison of gross linear kinetic and kinematic countermovement jump variables.

Jump Variables	Women		Men		*p*	*g*	ICC	%CV
	Mean	SD	Mean	SD				
Jump Height (cm)	24.3	4.7	32.1	5.1	<0.001	1.54	0.969	2.2
Movement Time (s)	0.752	0.136	0.812	0.077	0.069	0.53	0.685	7.4
RSImod (ratio)	0.34	0.10	0.40	0.10	0.040	0.58	0.899	6.1
Leg Stiffness (N·kg·m^−1^)	142.9	83.3	96.3	33.9	0.060	0.71	0.822	24.6
Eccentric COM Displacement (cm)	25.1	5.6	31.4	5.9	0.005	1.06	0.883	5.6
Concentric COM Displacement (cm)	34.4	5.4	42.2	6.2	0.001	1.30	0.914	3.9
Peak Eccentric Force (N·kg^−1^)	22.3	3.6	22.7	2.5	0.738	0.13	0.830	4.9
Peak Concentric Force (N·kg^−1^)	23.2	3.5	24.1	2.6	0.613	0.28	0.897	3.3
Peak Eccentric Power (W·kg^−1^)	14.5	4.1	16.2	4.0	0.287	0.41	0.755	9.2
Peak Concentric Power (W·kg^−1^)	41.8	6.7	50.3	6.6	0.001	1.24	0.965	2.0
Peak Eccentric Velocity (m·s^−1^)	1.02	0.19	1.19	0.21	0.001	0.82	0.914	4.7
Peak Concentric Velocity (m·s^−1^)	2.32	0.20	2.67	0.18	<0.001	1.79	0.972	0.9
Eccentric Impulse (N·kg^−1^·s)	1.04	0.19	1.21	0.21	0.041	0.82	0.835	4.7
Concentric Impulse (N·kg^−1^·s)	2.09	0.21	2.42	0.20	0.001	1.56	0.969	1.0

SD = Standard Deviation; ICC = Intraclass Correlation Coefficient; %CV = Percentage Coefficient of Variation; RSI_mod_ = Reactive Strength Index Modified; COM = Center of Mass.
